# Interaction analysis of ancestry-enriched variants with *APOE*-ɛ4 on MCI in the Study of Latinos-Investigation of Neurocognitive Aging

**DOI:** 10.1038/s41598-023-32028-2

**Published:** 2023-03-29

**Authors:** Einat Granot-Hershkovitz, Rui Xia, Yunju Yang, Brian Spitzer, Wassim Tarraf, Priscilla M. Vásquez, Richard B. Lipton, Martha Daviglus, Maria Argos, Jianwen Cai, Robert Kaplan, Myriam Fornage, Charles DeCarli, Hector M. Gonzalez, Tamar Sofer

**Affiliations:** 1 Division of Sleep and Circadian Disorders, Brigham and Women’s Hospital, 221 Longwood Avenue, Suite 225C, Boston, MA 02115 USA; 2 Department of Medicine, Harvard Medical School, Boston, MA USA; 3 Brown Foundation Institute of Molecular Medicine, McGovern Medical School, The University of Texas Health Science Center at Houston, Houston, TX USA; 4 Institute of Gerontology, Wayne State University, Detroit, MI USA; 5 Department of Urban Public Health, Charles R. Drew University of Medicine, Willowbrook, CA USA; 6 Albert Einstein College of Medicine, Montefiore Headache Center, Bronx, NY USA; 7 Institute for Minority Health Research, University of Illinois College of Medicine, Chicago, USA; 8 Division of Epidemiology and Biostatistics, University of Illinois at Chicago School of Public Health, Chicago, IL USA; 9 Department of Biostatistics, University of North Carolina at Chapel Hill, Chapel Hill, NC USA; 10 Department of Epidemiology and Population Health, Department of Pediatrics, Albert Einstein College of Medicine, Bronx, NY USA; 11 Division of Public Health Sciences, Fred Hutchinson Cancer Research Center, Seattle, WA USA; 12 Department of Neurology, Center for Neuroscience, University of California at Davis, Sacramento, CA USA; 13 Department of Neurosciences and Shiley-Marcos Alzheimer’s Disease Center, University of California, San Diego, La Jolla, CA USA; 14 Department of Biostatistics, Harvard T.H. Chan School of Public Health, Boston, MA USA

**Keywords:** Alzheimer's disease, Risk factors, Epidemiology, Genome-wide association studies

## Abstract

*APOE*-ɛ4 risk on Mild Cognitive Impairment (MCI) and Alzheimer’s Disease (AD) differs between race/ethnic groups, presumably due to ancestral genomic background surrounding the *APOE* locus. We studied whether African and Amerindian ancestry-enriched genetic variants in the *APOE* region modify the effect of the *APOE*-ɛ4 alleles on Mild Cognitive Impairment (MCI) in Hispanics/Latinos. We defined African and Amerindian ancestry-enriched variants as those common in one Hispanic/Latino parental ancestry and rare in the other two. We identified such variants in the *APOE* region with a predicted moderate impact based on the SnpEff tool. We tested their interaction with *APOE*-ɛ4 on MCI in the Study of Latinos-Investigation of Neurocognitive Aging (SOL-INCA) population and African Americans from the Atherosclerosis Risk In Communities (ARIC) study. We identified 5 Amerindian and 14 African enriched variants with an expected moderate effect. A suggestive significant interaction (*p*-value = 0.01) was found for one African-enriched variant, rs8112679, located in the *ZNF222* gene fourth exon. Our results suggest there are no ancestry-enriched variants with large effect sizes of interaction effects with *APOE*-ɛ4 on MCI in the *APOE* region in the Hispanic/Latino population. Further studies are needed in larger datasets to identify potential interactions with smaller effect sizes.

## Introduction

The *APOE*-ε4 allele is the strongest known genetic risk factor for MCI and Alzheimer’s Disease (AD)^1^. However, its effect on cognitive outcomes in Hispanics/Latinos is weaker and inconsistent compared to non-Hispanic Whites^2,3^. The genomes of Hispanics/Latinos are admixed, consisting of three predominant continental ancestries: Amerindian, African, and European^4^. In a recent publication, we performed an association study of *APOE* alleles and neurocognitive traits in middle-aged and older U.S. Hispanics from the SOL-INCA^5^. We discovered that their effects were modified by continental global genetic ancestry, e.g., Amerindian genetic ancestry protects from the risk conferred by *APOE*-ε4 on cognitive decline. Other studies conducted in admixed populations have also concluded that ancestry-specific genetic differences, either genome-wide or in the surrounding region of the *APOE* gene, modify the effect of *APOE* alleles on ADRD^6–9^. For example, a recent study explored missense variants in the *APOE* region and identified a variant coinherited with *APOE*-ε4 which mitigates the AD risk effect, and another variant coinherited with *APOE*-ε3, which has a protective effect^10^. Both variants were assessed in Europeans, due to the paucity of variant carriers in non-European ancestries. Another recent study identified a variant proximal to the *APOE* region, reducing the *APOE*-ε4 risk for AD in African Ancestry^9^.

We recently developed a computationally efficient method for ancestry-specific frequency estimation of bi-allelic genetic variants in a multi-way admixed population^11^. We published a database of ancestry-specific allele frequencies estimated from the HCHS/SOL Hispanic/Latino population. This unique dataset enables us to focus on ancestry-enriched genetic variants from African and Amerindian ancestries, that were previously understudied compared to European-enriched variants. We hypothesize that African and/or Amerindian ancestry-enriched genetic variants interact with the *APOE* alleles in their associations with AD and related cognitive outcomes, thus potentially explaining the modification of the global genetic ancestry on the effect of the *APOE* allele on cognitive outcomes^5^. Here, we study the modification effect of African and Amerindian ancestry-enriched genetic variants in the *APOE* region, on the effect of the *APOE*-ɛ4 alleles on MCI and MCI + , where MCI + defines a subset of the MCI group with suspected severe cognitive impairment, in the Study of Latinos-Investigation of Neurocognitive Aging (SOL-INCA) population. We focus on the enriched Amerindian and African variants in a region of 6Mbp encompassing the *APOE* gene. We further consider only variants with a moderate estimated effect based on SNPEff annotation. We conduct interaction analyses between the identified variants and the *APOE*-ε4 allele and its effect on MCI in SOL-INCA. We attempt replication of one suggestive interaction association between an African-enriched variant and *APOE*-ε4 allele on MCI in African Americans from the Atherosclerosis Risk In Communities (ARIC) study.

## Methods

### Study population

The HCHS/SOL is a population-based longitudinal multi-site cohort study of Hispanic/Latino adults in the U.S. that primarily enrolled participants from six self-identified backgrounds: Cuban, Central American, Dominican, Mexican, Puerto Rican, and South American^12,13^. A total of 16,415 adults, 18–74-year-old, were enrolled in the baseline visit at four field centers (Bronx, NY, Chicago, IL, Miami, FL, and San Diego, CA) (2008–2011). The baseline visit assessed cognitive function in 9,714 individuals aged 45 years or older. SOL-INCA is an ancillary study of HCHS/SOL, focusing on the middle-aged and older adult group who underwent cognitive assessment at visit 1. Overall, 6,377 individuals 50 or older with baseline cognitive testing participated in the SOL-INCA examination at or after HCHS/SOL visit 2, with an average of 7 years after the baseline exam. Of the 6,377 participants, 2140 were excluded from analyses (1701 did not consent for genetic data, 210 without *APOE* data, 76 had missing cognitive outcomes, and 153 had missing covariates and/or genetic variant reads), totalling an analytic sample of 4237 individuals.

The HCHS/SOL was approved by the institutional review boards (IRBs) at each field center, where all participants gave written informed consent in their preferred language (Spanish/English) to use their genetic and non-genetic data, and by the Non-Biomedical IRB at the University of North Carolina at Chapel Hill, to the HCHS/SOL Data Coordinating Center. All IRBs approving the study are: Non-Biomedical IRB at the University of North Carolina at Chapel Hill. Chapel Hill, NC; Einstein IRB at the Albert Einstein College of Medicine of Yeshiva University. Bronx, NY; IRB at Office for the Protection of Research Subjects (OPRS), University of Illinois at Chicago. Chicago, IL; Human Subject Research Office, University of Miami. Miami, FL; Institutional Review Board of San Diego State University, San Diego, CA. The study reported here was approved by the Mass General Brigham IRB under protocol #2019P000057. All methods and analyses of HCHS/SOL participants’ materials and data were carried out in accordance with human subject research guidelines and regulations.

### Neurocognitive outcome

Individuals were classified with MCI according to National Institute on Aging-Alzheimer’s Association criteria based on cognitive tests and self-reports^14^. Details about the SOL-INCA MCI diagnostic operational procedures have been previously published^15,16^. MCI was defined according to three criteria that had to be satisfied: (a) for any of the cognitive tests performed at the SOL-INCA exam, the score was lower than  −1 standard deviation (SD) of the mean, with means and SDs being defined based on SOL-INCA robust internal norms; (b) the rate of a global measure of yearly cognitive decline, estimated between the HCHS/SOL baseline and the SOL-INCA exam, was faster than −0.055 SD; (c) using the Everyday Cognition 12-item version (E-Cog12) questionnaire^17^ a participant self-reported subjective cognitive decline. The MCI group also included individuals classified as MCI + based on satisfying two conditions: (a) a cognitive test score lower than − 2 SD below the mean of any cognitive test performed at the SOL-INCA exam (with means and SDs based on SOL-INCA internal norms); (b) more than minimal impairment in self-reported instrumental activities of daily living (IADL)^18^. Individuals with MCI + were pooled together with the MCI individuals and together defined the MCI group.

### Genetic data

*APOE* genotyping was performed using commercial TaqMan assays previously described^19^. For individuals with missing *APOE* genotypes, we computed the genotypes based on phased whole-genome sequencing (WGS) data from TOPMed Freeze 8. Other genetic data were used based on genotyping (rather than WGS) using an Illumina custom array, previously reported^4^. Genome-wide imputation was conducted using the multi-ethnic NHLBI Trans-Omics for Precision Medicine (TOPMed) freeze 8 reference panel^20^. Principal components (PCs) were previously computed using PC-Relate^21^, and the kinship matrix was computed using the genetic data. ‘Genetic analysis groups’ were constructed based on a combination of self-identified Hispanic/Latino backgrounds and genetic similarity, and are classified as Central American, Cuban, Dominican, Mexican, Puerto Rican, and South American^4^.

### Ancestry enriched variants

We focused on a region of 6Mbp encompassing the *APOE* gene (~ 3Mbp from each side, mimicking the approach of Rajabli et al.^9^), chr19:42 Mb-48 Mb (GRCh38/hg38). Ancestry-specific frequencies of variants located in this region were calculated using GAFA^11^, a method we previously developed to estimate the frequencies of bi-allelic variants in admixed populations based on global proportions of genetic ancestries. Overall, the average ancestral global proportion of the three ancestries in the total dataset is 55% European, 30.5% Amerindian, and 14.5% African. Frequency estimation was based on n = 8933 HCHS/SOL individuals who consented to genetic data sharing with the broad scientific community. We defined Amerindian-enriched variants as those with both European and African minor allele frequency (MAF) < 0.01 and Amerindian frequency between 0.05 and 0.95. Using the same principle, we defined African-enriched variants. MAF is computed in a population, and it quantifies how likely it is for an individual from the population to have a specific genetic variant. A variant with a very low MAF (often MAF < 0.01 is considered rare) in a population is likely to be observed only in a few individuals in that population, and a variant with a high MAF (the largest possible value is 0.5) is likely to be observed in many individuals. Thus, an Amerindian (African) enriched variant is likely to be observed almost only in individuals who inherited the corresponding genomic region from an Amerindian (African) ancestor.

### Bioinformatics

We performed bioinformatics analyses using publicly available databases and tools for the ancestry-enriched variants. Variant data were annotated using SnpEff (V.4.3). The SnpEff software takes as an input a genetic data file (variant calls on a VCF file) and it annotates each of the variants in the file using sequence ontology terms of predicted effects of the variants on known genes (e.g., codon deletion, exon duplication). It also provides impact prediction, with four categories: high, moderate, low, and modifier. Moderate impact means that the variant might change protein effectiveness, and high impact means that the variant is assumed to have high disruptive impact on the relevant protein. Low impact is annotated for variants that assumed to be harmless, and modifier impact is usually assigned for non-coding variants or variants affecting non-coding genes, where there is no evidence of impact. Variants with estimated moderate putative impact were selected for further analyses. We further annotated the variants by using additional tools: RegulomeDB^22^, GTEx Portal, GWAS catalog^23^, and Phegen^24^. Finally, we computed the MAF and minor allele counts (MAC) of each of the selected variants in the six Hispanic genetic analysis groups.

### Statistical analysis

We provided descriptive statistics to characterize the demographic and cognitive outcome and *APOE* alleles distributions in the analytic dataset of n = 4,237 individuals. For each ancestry-enriched variant with a SnpEff predicted moderate impact, we tested the interaction associations between the variant and *APOE*-ε4 allele on MCI. Models included the variant, the *APOE*-ε4 allele (additive mode), and the interaction term of the variant with the *APOE*-ε4 allele. We used the complex survey design from the R ‘survey’ package^25^, with a “quasipoisson” family for binary traits. This method accounts for the stratification, clustering, and probability weighting in HCHS/SOL to allow correct generalizations to the HCHS/SOL target population. Models were adjusted for age, sex, education, center, first 5 PCs of genetic data, and genetic analysis group. The significance of the results was evaluated in two ways, to protect from potential high type 1 error due to the low proportion of *APOE* variant and the enriched variant alleles. First, through 5000 permutations, we performed multivariant Wald tests to jointly test the significance of the variant and variant-*APOE*-ε4 allele interaction. We also performed multivariant Wald tests to jointly test the association of the variant and variant-*APOE*-ε4 allele interaction and the *APOE*-ε4 allele. Second, we used mixed models and the BinomiRare test for low-count variants to test the association of the variant and variant-*APOE*-ε4 allele interaction^26^. Mixed models used correlation matrices to account for genetic relatedness (kinship), household, and block unit sharing as random effects, and were implemented, along with the BinomiRare, in the GENESIS R/Bioconductor package^27^, version 3.15.

### Estimation of interaction associations with MCI in the ARIC study

We further evaluated the interaction associations between the African-enriched variant and *APOE*-ε4 in African Americans from the Atherosclerosis Risk in Communities (ARIC) study. ARIC is a longitudinal cohort study with genetic and cognitive measures^28,29^. The protocol for MCI/dementia diagnosis in ARIC has been previously described^30^ and is provided in Supplementary Note 1. Data from ARIC Visit 5 were used in this analysis. Further details are provided in Supplementary Note 1.

Similar to the model in SOL-INCA, the statistical model in ARIC included adjustment for age, sex, education, 5 PCs, and study-site, and tested the interaction associations between the variant and *APOE*-ε4 allele on a neurocognitive outcome. We also performed multivariant Wald tests as described above for SOL-INCA.

## Results

Table 1 characterizes the demographic, health, and lifestyle characteristics of the SOL-INCA analytic dataset (n = 4237). Overall, around 52% of the participants are females, with a weighted mean age of 62 years at the SOL-INCA visit. MCI prevalence is ~ 11.3%. *APOE*-ε3 is the most frequent allele, with 0.83 allele frequency, while alleles 4 and 2 are relatively rare (frequencies of 0.12 and 0.049 respectively). Fifty-eight of the individuals with MCI (1.4% of the sample) were classified as having suspected severe impairment (MCI +).

**Table 1:** Demographics, genetic and neurocognitive characteristics of SOL-INCA analytic sample.

Characteristic		
All N		4237
Sex (%)	Female	2649 (52.4)
Age in years Mean (SD)		62.10 (8.19)
Education (%)		
	< 12	1637 (35.7)
	12	922 (21.9)
	> 12	1678 (42.3)
Background		
	Central American	420 (7.1)
	Cuban	909 (32.2)
	Dominican	425 (10.0)
	Mexican	1,411 (28.1)
	Puerto Rican	753 (16.6)
	South American	319 (6.0)
*APOE* genotype (%)		
	ε2/ε2	13 (0.3)
	ε2/ε3	338 (7.9)
	ε2/ε4	51 (1.3)
	ε3/ε3	2927 (69.5)
	ε3/ε4	837 (19.3)
	ε4/ε4	71 (1.7)
*APOE* allele (%)		
	ε2	415 (4.9)
	ε3	7029 (83.1)
	ε4	1030 (12)
Neurocognitive trait		
MCI* (%)		448 (11.3)

SOL-INCA: Study of Latinos-Investigation of Neurocognitive Aging (SOL-INCA); SD: standard deviation; MCI: mild cognitive impairment. (%) based on the sampling weights and complex survey design.

*The MCI group includes 58 individuals with suspect severe impairment (MCI +).

### Ancestry enriched variants

In the 6Mbp encompassing the *APOE* gene (~ 3Mbp from each side), chr19:42–48 Mb (GRCh38/hg38), we identified 260 Amerindian-enriched variants in the HCHS/SOL study population, with ≥ 5% frequency for Amerindian, and ≤ 1% frequency for African and European ancestries (Supplementary Table 1). Similarly, we identified 798 African-enriched variants in the HCHS/SOL study population (Supplementary Table 2). Using SnpEff (V.4.3) variant annotation, we selected 5 Amerindian- and 14 African-enriched variants, with a predicted moderate putative impact, for further interaction analyses with *APOE*-ε4 on MCI. Annotations of the variants and their estimated ancestral frequencies are presented in Table 2. Eighteen out of the total 19 selected enriched variants are missense variants. According to the GTExportal, 11 out of the 14 selected African-enriched variants have previously been associated with gene expressions in various tissues, i.e., they are expression quantitative trait loci (eQTLs) in these tissue, including brain cerebellum tissue.

**Table 2:** Annotation of the ancestry-enriched genetic variants used for interaction analysis in SOL-INCA analytic dataset.

rsID	Position (hg38)	A1	A2	HCHS/SOL ancestry specific frequency			SNPeff		RegulomeDB variant classification		GTEx portal eQTL (the strongest gene-tissue) association		
				AFR	EUR	AMR	Annotation	Gene Name	Rank*	Score**	Gene Symbol	*P*-Value	Tissue
rs148194580	chr19:43,612,510	G	A	0.004	0.002	0.126	missense_variant	*SRRM5*	4	0.609	–	–	–
rs76261208	chr19:44,010,922	C	A	0.009	0.006	0.368	missense_variant	*ZNF230*	5	0.590	–	–	–
rs142133384	chr19:44,304,792	G	C	0.005	0.006	0.187	missense_variant	*ZNF235*	4	0.705	–	–	–
rs143674072	chr19:45,510,425	C	T	0.004	0.002	0.186	sequence_feature	*VASP*	4	0.609	–	–	–
rs150300804	chr19:47,726,147	C	T	0.004	0.007	0.068	missense_variant	*EHD2*	4	0.609	–	–	–
rs3745238	chr19:42,426,500	C	T	0.132	0.003	0.006	missense_variant	*LIPE*	4	0.609	*LIPE-AS1*	3.30E−10	Testis
rs16975748	chr19:42,426,621	A	T	0.132	0.003	0.006	missense_variant	*LIPE*	4	0.609	*LIPE-AS1*	3.30 E−10	Testis
rs16975750	chr19:42,426,852	A	G	0.245	0.005	0.008	missense_variant	*LIPE*	4	0.609	*CXCL17*	2.30 E−10	Esophagus—Mucosa
rs8111171	chr19:42,527,362	G	T	0.407	0.003	0.008	missense_variant	*CEACAM1*	4	0.609	*CXCL17*	5.20 E−11	Esophagus—Mucosa
rs8103051	chr19:42,583,276	A	C	0.218	0.004	0.008	missense_variant	*CEACAM8*	5	0.135	*CXCL17*	6.60 E−14	Esophagus—Mucosa
rs28367882	chr19:42,594,779	C	A	0.212	0.004	0.008	missense_variant	*CEACAM8*	4	0.609	*CXCL17*	6.60 E−14	Esophagus—Mucosa
rs111674083	chr19:42,755,012	A	C	0.124	0.002	0.007	missense_variant	*PSG8*	7	0.184	*LIPE-AS1*	4.20 E−08	Testis
rs76352186	chr19:42,764,090	C	T	0.213	0.009	0.004	missense_variant	*PSG8*	5	0.135	*GRIK5*	4.70 E−08	Testis
rs140974685	chr19:42,916,334	G	C	0.113	0.002	0.004	missense_variant	*PSG6*	3a	0.354	*–*	–	–
rs28477226	chr19:43,461,652	G	T	0.095	0.003	0.004	missense_variant	*LYPD3*	4	0.609	*SRRM5*	3.70 E−06	Liver
rs8112679	chr19:44,032,462	G	A	0.104	0.006	0.004	missense_variant	*ZNF222*	4	0.609	*–*	–	–
rs35365841	chr19:44,657,802	G	A	0.185	0.002	0.006	missense_variant	*PVR*	4	0.609	*PVR*	2.90 E−06	Brain—Cerebellum
rs12460007	chr19:45,152,811	G	T	0.070	0.005	0.005	missense_variant	*NKPD1*	4	0.705	*–*	–	–
rs309195	chr19:47,066,585	G	C	0.450	0.002	0.010	missense_variant	*ZC3H 4*	3a	0.445	*DHX34*	2.40 E−05	Brain—Cerebellar Hemisphere

A1 is the effect allele, also the minor allele. A2 is the other (non-effect) allele.

AFR: African, EUR: European, AMR: Amerindian ancestries.

For all variants in the table, SNPeff putative impact is “moderate”.

* Category based scoring system (class from 1–7, with lower rank means higher functional support):

3a Transcription factor (TF) binding + any motif + DNase peak.

4 TF binding + DNase peak.

5 TF binding or DNase peak.

**Lower scores indicate increasing evidence for a variant to be located in a functional region.

Supplementary Table 3 provides the computed MAF and MAC of each of the selected variants across the genetic analysis groups corresponding to the six Hispanic backgrounds. As expected, Amerindian-enriched variants tended to be more common in groups with high Amerindian ancestry: Central and South American, and Mexican individuals, while African-enriched variants were more common in the Caribbean group, that have higher African ancestry: Dominican, Cuban, and Puerto Rican.

### Interaction association between ancestry enriched variants and *APOE* alleles on MCI

Results of the enriched Amerindian or African variants’ interaction with *APOE*-ɛ4 on MCI analysis based on the 5,000 permutations multivariant Wald tests are reported in Table 3. No statistically significant interaction associations were identified. However, results of the enriched Amerindian or African variants’ interaction with *APOE*-ɛ4 on MCI analysis, the BinomiRare test identified one nominally significant interaction between an African enriched variant, rs8112679, and *APOE*-ɛ4 on MCI (*p*-value = 0.017) (Table 4).

**Table 3:** Permutation results (n = 5,000 permutations) for the ancestry-enriched genetic variants and interaction associations between the variants and *APOE* alleles on MCI in SOL-INCA analytic dataset.

rsID	Position (hg38) chr19	A1	A2	*APOE*-ɛ4		variant		*APOE*-ɛ4-variant interaction		Joint analyses	
				OR	*p*-value	OR	*p*-value	OR	*p*-value	p-value, variant + interaction variant-*APOE*-ɛ4	*p*-value, variant + *APOE*-ɛ4 + interaction variant-*APOE*-ɛ4
Amerindian-enriched variants											
rs148194580	43,612,510	A	G	0.916	0.795	0.279	0.599	3.126	0.481	0.296	0.424
rs76261208	44,010,922	A	C	0.915	0.893	2.686	0.242	7.263	0.060	0.559	0.654
rs142133384	44,304,792	C	G	0.855	0.802	1.001	0.897	3.149	0.553	0.418	0.525
rs143674072	45,510,425	T	C	0.783	0.546	0.188	0.197	0.344	0.312	0.245	0.355
rs150300804	47,726,147	T	C	0.950	0.563	6.289	0.673	0.280	0.523	0.784	0.836
African-enriched variants											
rs3745238	42,426,500	T	C	0.821	0.667	0.424	0.283	0.878	0.756	0.888	0.871
rs16975748	42,426,621	T	A	0.821	0.667	0.424	0.283	0.878	0.756	0.888	0.871
rs16975750	42,426,852	G	A	0.820	0.751	2.045	0.865	1.600	0.832	0.816	0.773
rs8111171	42,527,362	T	G	0.921	0.920	0.517	0.596	2.989	0.461	0.571	0.563
rs8103051	42,583,276	C	A	0.869	0.840	0.830	0.746	2.450	0.500	0.998	0.909
rs28367882	42,594,779	A	C	0.868	0.858	0.894	0.858	2.554	0.465	0.994	0.910
rs111674083	42,755,012	C	A	0.825	0.624	0.943	0.615	0.440	0.571	0.507	0.567
rs76352186	42,764,090	T	C	1.100	0.973	0.803	0.826	7.452	0.121	0.523	0.622
rs140974685	42,916,334	C	G	0.792	0.758	7.591	0.463	2.366	0.723	0.621	0.675
rs28477226	43,461,652	T	G	0.808	0.745	1.843	0.883	0.867	0.808	0.923	0.878
rs8112679	44,032,462	A	G	0.830	0.676	3.634	0.565	0.898	0.970	0.834	0.836
rs35365841	44,657,802	A	G	0.844	0.598	0.928	0.562	0.300	0.676	0.780	0.820
rs12460007	45,152,811	T	G	1.014	0.412	2.411	0.476	10.948	0.080	0.746	0.781
rs309195	47,066,585	C	G	0.829	0.840	3.741	0.293	4.737	0.172	0.162	0.214

A1 is the effect allele, also the minor allele. A2 is the other (non-effect) allele.

OR: odds ratio. Note that variant and interaction ORs are often high due to high variability caused by low count of the rare allele.

**Table 4:** BinomiRare tests for the ancestry-enriched genetic variants and interaction associations between the variants and *APOE-e4* allele on MCI in SOL-INCA analytic dataset.

rsID	Position (hg38) chr19	A1	A2	Variant					Variant interaction with APOE-e4 allele				
				n carrier*	nD carrier**	Expected nD carrier***	p-value	Mid-pval	n carrier*	nD carrier**	Expected nD carrier***	p-value	Mid-p-value
Amerindian-enriched variants													
rs148194580	43,612,510	A	G	293	30	30.39	4.96 E−01	4.67 E−01	75	7	8.00	7.03 E−01	6.37 E−01
rs76261208	44,010,922	A	C	830	91	87.58	7.75 E−01	7.53 E−01	168	16	19.51	1.00E + 00	9.51 E−01
rs142133384	44,304,792	C	G	456	51	47.91	4.87 E−01	4.63 E−01	107	13	12.00	2.77 E−01	2.46 E−01
rs143674072	45,510,425	T	C	368	33	37.48	6.63 E−01	6.32 E−01	77	10	8.51	1.00E + 00	9.27 E−01
rs150300804	47,726,147	T	C	204	23	21.63	4.89 E−01	4.54 E−01	50	10	6.31	1.00E + 00	9.14 E−01
African-enriched variants													
rs3745238	42,426,500	T	C	197	21	22.00	8.19 E−01	7.75 E−01	65	8	7.60	3.30 E−01	2.83 E−01
rs16975748	42,426,621	T	A	197	21	22.00	8.19 E−01	7.75 E−01	65	8	7.60	3.30 E−01	2.83 E−01
rs16975750	42,426,852	G	A	342	42	37.07	5.97 E−01	5.68 E−01	109	13	12.50	2.86 E−01	2.54 E−01
rs8111171	42,527,362	T	G	527	50	56.32	6.69 E−01	6.43 E−01	173	16	19.62	3.97 E−01	3.64 E−01
rs8103051	42,583,276	C	A	320	37	35.03	4.69 E−01	4.42 E−01	108	11	12.59	1.74 E−01	1.50 E−01
rs28367882	42,594,779	A	C	311	36	33.74	3.12 E−01	2.90 E−01	108	11	12.59	1.74 E−01	1.50 E−01
rs111674083	42,755,012	C	A	188	20	20.34	2.84 E−01	2.60 E−01	63	10	7.15	4.22 E−01	3.67 E−01
rs76352186	42,764,090	T	C	320	27	33.92	8.54 E−01	8.19 E−01	82	3	9.34	1.12 E−01	9.44 E−02
rs140974685	42,916,334	C	G	142	20	16.06	4.21 E−01	3.84 E−01	47	8	5.30	9.97 E−02	7.86 E−02
rs28477226	43,461,652	T	G	130	16	14.90	1.00E + 00	9.45 E−01	43	5	5.77	1.07 E−01	8.63 E−02
rs8112679	44,032,462	A	G	165	21	17.78	4.45 E−01	4.11 E−01	51	5	6.31	**1.65** E−**02**	**1.25** E−**02**
rs35365841	44,657,802	A	G	258	24	27.31	7.58 E−01	7.21 E−01	86	7	10.32	9.03 E−02	7.70 E−02
rs12460007	45,152,811	T	G	124	12	13.09	4.60 E−01	4.18 E−01	36	5	4.40	1.00E + 00	8.98 E−01
rs309195	47,066,585	C	G	596	68	64.09	7.89 E−01	7.64 E−01	194	22	22.85	9.11 E−01	8.66 E−01

A1 is the effect allele, also the minor allele. A2 is the other (non-effect) allele.

*n carrier: number of individuals with the rare variant allele, or number of individuals with both the rare allele and APOE-e4 allele (for the interaction term).

**nD carrier: number of individuals with the rare variant allele (or both the rare allele and APOE-e4 allele for the interaction term) who also have MCI.

***expected nD carrier: expected number of individuals to have MCI among the carriers, based on the probability of MCI that was estimated without accounting for the variant (or for the interaction).

### Estimation of interaction associations with MCI in the ARIC study and meta-analysis

Supplementary Table 4 characterizes the demographic, cognitive outcome, and *APOE* alleles’ distributions in the ARIC African Americans analytic dataset. The African enriched variant’s interaction (rs8112679) with the *APOE*-ɛ4 on MCI was not significant in the ARIC African Americans analytic dataset (Supplementary Table 5).

A replication attempt of the interaction association between the African variant previously reported by Rajabli et al.^9^ and the *APOE*-Ɛ4 allele on MCI in the SOL-INCA analytic dataset did not present a significant result (Supplementary Table 6).

## Discussion

In this study, we leveraged recently published data on ancestry-specific genetic variant frequencies in the Hispanic/Latino population^11^, to explore the interaction effects of African and Amerindian ancestry-enriched genetic variants with *APOE*-ɛ4 on MCI in the Hispanic/Latino US population. We found suggestive evidence for an interaction effect of an African-enriched variant, rs8112679, with *APOE*-ɛ4 on MCI, with the minor allele A having a protective effect on MCI. This result did not replicate in the ARIC African American analytic sample. Rs8112679 is a missense variant, located in exon 4 of the *ZNF222* gene. *ZNF222* gene is predicted to be involved in the regulation of transcription by RNA polymerase II. A previous study suggests the involvement of *ZNF222* in late-onset Alzheimer’s disease^31^. Hopefully, future studies, particularly with African and African American individuals in which the variant is common and, ideally, a familial study with individuals both affected and unaffected by MCI or AD, would follow up on this variant and study its presence in affected individuals.

Our study is based on relatively small sample sizes therefore its statistical power is limited for the association of low-frequency and rare variants, all the more so for interaction analyses. Only large effect sizes could have been discovered. Our results suggest there are no large effect sizes of ancestry-enriched variants interacting with *APOE*-ɛ4 on MCI in the *APOE* region in the Hispanic/Latino population. Further analysis with larger sample sizes, and meta-analyses with additional studies, are needed to identify ancestry-enriched variants interacting with *APOE*-ɛ4 on MCI. It would also be interesting to increase the region in which variants are considered, potentially within other known AD genes or genome-wide. The major limitation is power, reduced by high multiple testing burden. Collaboration between multiple studies with diverse individuals with Amerindian and/or African ancestry will be critical. Another limitation of our study is that a subset of the MCI individuals was classified as MCI + , who fell into a gray zone between MCI and dementia. That is, their cognitive scores or functional abilities (i.e., IADLs) did not meet strict criteria for MCI or dementia. As SOL-INCA study population grows older, we will know whether the MCI and MCI + individuals convert to dementia. The cognitive aging trajectories of MCI is important and unanswered research question facing the field. It is possible that genetic determinants underlying mild and severe cognitive impairment are different. However, larger datasets are required to study this hypothesis, to assess trajectories of mild and severe cognitive impairment, and to identify trait-specific genetic basis.

Identification of variants interacting with *APOE*-ɛ4 may further delineate the role of APOE in the pathogenesis of MCI and AD and advance novel therapeutics. It may also lead to population-specific risk predictions and help reduce health disparities in the general population.

## Supplementary Information

Supplementary Information 1.

Supplementary Tables.

## Data Availability

HCHS/SOL genetic and phenotypic data can be obtained through the study's Data Coordinating Center using an approved data use agreement. Information is provided in https://sites.cscc.unc.edu/hchs/. HCHS/SOL genetic and phenotypic data can also be obtained from dbGaP under accession number phs000810.v1.p1. ARIC genetic and phenotypic data can be obtained through the study's Data Coordinating Center using an approved data use agreement. Information is provided in https://sites.cscc.unc.edu/aric/distribution-agreements.

## References

[1] Tervo S, Kivipelto M, Hänninen T (2004). Incidence and risk factors for mild cognitive impairment: a population-based three-year follow-up study of cognitively healthy elderly subjects. Dement. Geriatr. Cogn. Disord..

[2] Alzheimer Association (2018). Alzheimer's disease facts and figures. Alzheimer's & Dementia.

[3] Dilworth-Anderson P, Hendrie HC, Manly JJ, Khachaturian AS, Fazio S (2008). Diagnosis and assessment of Alzheimer's disease in diverse populations. Alzheimers Dement..

[4] Conomos MP, Laurie CA, Stilp AM (2016). Genetic diversity and association studies in US Hispanic/Latino populations: applications in the Hispanic community health study/study of Latinos. Am. J. Human Genet..

[5] Granot-Hershkovitz E, Tarraf W, Kurniansyah N (2020). APOE alleles' association with neurocognitive function differ across Hispanic background groups. Alzheimers Dement..

[6] Hohman TJ, Cooke-Bailey JN, Reitz C (2016). Global and local ancestry in African–Americans: implications for Alzheimer's disease risk. Alzheimers Dement..

[7] Rajabli F, Feliciano BE, Celis K (2018). Ancestral origin of ApoE ε4 Alzheimer disease risk in Puerto Rican and African American populations. PLoS Genet..

[8] Blue EE, Horimoto A, Mukherjee S, Wijsman EM, Thornton TA (2019). Local ancestry at APOE modifies Alzheimer's disease risk in Caribbean Hispanics. Alzheimers Dement..

[9] Rajabli F, Beecham GW, Hendrie HC (2022). A locus at 19q1331 significantly reduces the ApoE ε4 risk for Alzheimer's Disease in African Ancestry. PLoS Genet..

[10] Le Guen Y, Belloy ME, Grenier-Boley B (2022). Association of rare APOE missense variants V236E and R251G With risk of Alzheimer disease. JAMA Neurol..

[11] Granot-Hershkovitz E, Sun Q, Argos M (2022). AFA: ancestry-specific allele frequency estimation in admixed populations: the Hispanic community health study/study of Latinos. HGG Adv..

[12] Lavange LM, Kalsbeek WD, Sorlie PD (2010). Sample design and cohort selection in the Hispanic community health study/study of Latinos. Ann. Epidemiol..

[13] Sorlie PD, Aviles-Santa LM, Wassertheil-Smoller S (2010). Design and implementation of the Hispanic community health study/study of Latinos. Ann. Epidemiol..

[14] Albert MS, DeKosky ST, Dickson D (2011). The diagnosis of mild cognitive impairment due to Alzheimer's disease: recommendations from the National Institute on Aging-Alzheimer's Association workgroups on diagnostic guidelines for Alzheimer's disease. Alzheimers Dement..

[15] González HM, Tarraf W, Fornage M (2019). A research framework for cognitive aging and Alzheimer's disease among diverse US Latinos: design and implementation of the Hispanic Community Health Study/Study of Latinos-Investigation of Neurocognitive Aging (SOL-INCA). Alzheimers Dement..

[16] González HM, Tarraf W, Schneiderman N (2019). Prevalence and correlates of mild cognitive impairment among diverse hispanics/latinos: Study of Latinos-Investigation of Neurocognitive Aging results. Alzheimers Dement..

[17] Tomaszewski Farias S, Mungas D, Harvey DJ, Simmons A, Reed BR, Decarli C (2011). The measurement of everyday cognition: development and validation of a short form of the Everyday Cognition scales. Alzheimers Dement..

[18] González HM, Tarraf W, Gouskova N (2015). Neurocognitive function among middle-aged and older Hispanic/Latinos: results from the Hispanic Community Health Study/Study of Latinos. Arch. Clin. Neuropsychol..

[19] González HM, Tarraf W, Jian X (2018). Apolipoprotein E genotypes among diverse middle-aged and older Latinos: Study of Latinos-Investigation of Neurocognitive Aging results (HCHS/SOL). Sci. Rep..

[20] Kowalski MH, Qian H, Hou Z (2019). Use of >100,000 NHLBI Trans-Omics for Precision Medicine (TOPMed) Consortium whole genome sequences improves imputation quality and detection of rare variant associations in admixed African and Hispanic/Latino populations. PLoS Genet..

[21] Conomos MP, Reiner AP, Weir BS, Thornton TA (2016). Model-free estimation of recent genetic relatedness. Am. J. Human Genet..

[22] Boyle AP, Hong EL, Hariharan M (2012). Annotation of functional variation in personal genomes using RegulomeDB. Genome Res..

[23] Buniello A, MacArthur JAL, Cerezo M (2019). The NHGRI-EBI GWAS Catalog of published genome-wide association studies, targeted arrays and summary statistics 2019. Nucleic Acids Res..

[24] Ramos EM, Hoffman D, Junkins HA (2014). Phenotype-Genotype Integrator (PheGenI): synthesizing genome-wide association study (GWAS) data with existing genomic resources. Eur. J. Human Genet..

[25] Lumley T, Scott A (2014). Tests for regression models fitted to survey data. Aust. N. Z. J. Stat..

[26] Sofer T, Lee J, Kurniansyah N, Jain D, Laurie CA, Gogarten SM, Conomos MP, Heavner B, Yao H, Kooperberg C, Haessler J, Vasan RS, Adrienne Cupples L, Coombes BJ, Seyerle A, Gharib SA, Chen H, O’Connell JR, Zhang M, Gottlieb DJ, Psaty BM, Longstreth WT, Rotter JI, Taylor KD, Rich SS, Guo X, Boerwinkle E, Morrison AC, Pankow JS, Johnson AD, Pankratz N, Reiner AP, Redline S, Smith NL, Rice KM, Schifano ED (2021). BinomiRare: a robust test for association of a rare genetic variant with a binary outcome for mixed models and any case-control proportion. Human Genet. Genom. Adv..

[27] Gogarten SM, Sofer T, Chen H, Chaoyu Y, Brody JA, Thornton TA, Rice KM, Conomos MP (2019). Genetic association testing using the GENESIS R/Bioconductor package. Bioinformatics.

[28] The ARIC investigators (1989). The atherosclerosis risk in communities (ARIC) study: design and objectives. Am. J. Epidemiol..

[29] Wright JD, Folsom AR, Coresh J (2021). The ARIC (atherosclerosis risk in communities) study: JACC focus seminar 3/8. J. Am. Coll. Cardiol..

[30] Knopman DS, Gottesman RF, Sharrett AR (2016). Mild cognitive impairment and dementia prevalence: the atherosclerosis risk in communities neurocognitive study (ARIC-NCS). Alzheimers Dement. (Amst).

[31] Rao S, Ghani M, Guo Z (2018). An APOE-independent cis-eSNP on chromosome 19q13.32 influences tau levels and late-onset Alzheimer's disease risk. Neurobiol. Aging.

