# Supplementary material for: Interaction analysis of ancestry-enriched variants with APOE-ɛ4 on MCI in the Study of Latinos-Investigation of Neurocognitive Aging
Source: Sci Rep. 2023 Mar 29;13:5114. doi: 10.1038/s41598-023-32028-2 (PMC10060219; doi:10.1038/s41598-023-32028-2)
Supplement: Supplementary file 1 — Supplementary Information 1. [file 41598_2023_32028_MOESM1_ESM.docx]

# Supplementary Note 1: ARIC Methods

The Atherosclerosis Risk in Communities (ARIC) study is a prospective longitudinal study of the development of atherosclerosis, and its clinical sequelae in which 15,792 individuals aged 45-64 years from four communities in the United States were enrolled at the baseline examination (1987-1989). A detailed description of the ARIC study has been reported previously^1^. Various medical information and specimens were collected at each of the eight examinations. Written informed consent was provided by all study participants, and the study design and methods were approved by the institutional review boards at each of the collaborating medical institutions: University of Mississippi Medical Center Institutional Review Board (Jackson Field Center); Wake Forest University Health Sciences Institutional Review Board (Forsyth County Field Center); University of Minnesota Institutional Review Board (Minnesota Field Center); and Johns Hopkins University School of Public Health Institutional Review Board (Washington County Field Center). In this analysis, we use 3 1,032 African American ARIC participants with Mild Cognitive Impairment (MCI) status determined based on data from ARIC visit 5. Detailed descriptions of participants' health, demographic, and lifestyle characteristics are presented in Supplementary Table 3.

The protocol for MCI and dementia diagnosis in ARIC has been previously described in detail^2^. At visit 5, all participants were identified as cognitively normal or possible MCI/dementia cases with the use of an algorithm that considered MMSE scores, Clinical Dementia Rating sum of boxes, concurrent performance on the neuropsychological test battery, and change in cognitive function from previous assessments. All cases that were identified as possible MCI/dementia by this algorithm and a sample of cognitively normal participants were reviewed by 2 experts (a physician and a neuropsychologist), who classified participants' cognitive status as normal, MCI, or dementia, with discordant diagnoses adjudicated by a third reviewer.

## References

1. Wright JD, Folsom AR, Coresh J, et al. The ARIC (Atherosclerosis Risk In Communities) Study: JACC Focus Seminar 3/8. J Am Coll Cardiol 2021;77:2939-2959.

2. Knopman DS, Gottesman RF, Sharrett AR, et al. Mild Cognitive Impairment and Dementia Prevalence: The Atherosclerosis Risk in Communities Neurocognitive Study (ARIC-NCS). Alzheimers Dement (Amst) 2016;2:1-11.
